# Microbial Community of High Arsenic Groundwater in Agricultural Irrigation Area of Hetao Plain, Inner Mongolia

**DOI:** 10.3389/fmicb.2016.01917

**Published:** 2016-12-06

**Authors:** Yanhong Wang, Ping Li, Zhou Jiang, Aki Sinkkonen, Shi Wang, Jin Tu, Dazhun Wei, Hailiang Dong, Yanxin Wang

**Affiliations:** ^1^State Key Laboratory of Biogeology and Environmental Geology, China University of GeosciencesWuhan, China; ^2^School of Environmental Studies, China University of GeosciencesWuhan, China; ^3^Department of Environmental Sciences, University of HelsinkiLahti, Finland; ^4^Lawrence Berkeley National Laboratory, BerkeleyCA, USA; ^5^Department of Geology and Environmental Earth Science, Miami University, OxfordOH, USA

**Keywords:** arsenic, groundwater, irrigation, microbial community, illumina MiSeq, Hetao Plain

## Abstract

Microbial communities can play important role in arsenic release in groundwater aquifers. To investigate the microbial communities in high arsenic groundwater aquifers in agricultural irrigation area, 17 groundwater samples with different arsenic concentrations were collected along the agricultural drainage channels of Hangjinhouqi County, Inner Mongolia and examined by illumina MiSeq sequencing approach targeting the V4 region of the 16S rRNA genes. Both principal component analysis and hierarchical clustering results indicated that these samples were divided into two groups (high and low arsenic groups) according to the variation of geochemical characteristics. Arsenic concentrations showed strongly positive correlations with ${\mathrm{NH}}_{4}^{+}$ and total organic carbon (TOC). Sequencing results revealed that a total of 329–2823 operational taxonomic units (OTUs) were observed at the 97% OTU level. Microbial richness and diversity of high arsenic groundwater samples along the drainage channels were lower than those of low arsenic groundwater samples but higher than those of high arsenic groundwaters from strongly reducing areas. The microbial community structure in groundwater along the drainage channels was different from those in strongly reducing arsenic-rich aquifers of Hetao Plain and other high arsenic groundwater aquifers including Bangladesh, West Bengal, and Vietnam. *Acinetobacter* and *Pseudomonas* dominated with high percentages in both high and low arsenic groundwaters. *Alishewanella, Psychrobacter, Methylotenera*, and *Crenothrix* showed relatively high abundances in high arsenic groundwater, while *Rheinheimera* and the unidentified OP3 were predominant populations in low arsenic groundwater. Archaeal populations displayed a low occurrence and mainly dominated by methanogens such as *Methanocorpusculum* and *Methanospirillum*. Microbial community compositions were different between high and low arsenic groundwater samples based on the results of principal coordinate analysis and co-inertia analysis. Other geochemical variables including TOC, ${\mathrm{NH}}_{4}^{+}$, oxidation-reduction potential, and Fe might also affect the microbial composition.

## Introduction

Arsenic (As)-contaminated groundwater used for drinking water has adversely impact the health of more than 140 million people all over the world including Bangladesh, Vietnam, India, China, Chile, USA, and Argentina (Berg et al., 2001; Anawar et al., 2011; Nicolli et al., 2012; Ayotte et al., 2014; Guo et al., 2014). Many studies have been conducted in recent years to investigate the geochemistry, biochemistry, and microbial ecology of As in groundwater aquifers (Sarkar et al., 2014; Wang et al., 2014b, 2015) and the previous results showed that As release and mobilization were usually controlled by a series of microbially mediated reactions and geochemical processes. As one kind of important geological mediators, microorganisms can utilize As as an electron acceptor/donor or energy source, thereby changing the As speciation and mineralization in groundwater aquifers (Slyemi and Bonnefoy, 2012; Amend et al., 2014; Ghosh et al., 2014). To date, more than 200 strains assigned to 11 phyla of bacteria and one phylum of archaea (Crenarchaeota) have been isolated from different environmental conditions such as groundwater, hot springs, marine sediments, and mine drainage (Amend et al., 2014). Besides, some functional populations such as Fe-reducing bacteria and sulfate reducing bacteria have been previously found in As-rich aquifers (Kirk et al., 2010; Li et al., 2014, 2015). Recent studies showed that agricultural activities such as irrigation might play important roles in As release and mobilization. The input of organic matter and fertilizers during the drainage process could change the environmental conditions, which could further affect the microbial communities (Kefala-Agoropoulou et al., 2008; Mayorga et al., 2013). These studies showed the importance of microorganisms in As release and mobilization, however, failed to provide information of *in situ* microbial community structure which is important in understanding the interaction between microorganism and As geochemistry in groundwater in agricultural area.

The Hetao Plain is one of the most serious and representative As-threatened areas in northwest China, with over 300,000 residents vulnerable to arsenicosis (Guo et al., 2003). Arsenic in groundwater in the Hetao Plain is released primarily through natural processes and anthropogenic activities including agricultural irrigation and mining (Deng, 2008; Guo et al., 2008). Our previous studies have demonstrated microbial community structure and functional microbial populations in high As groundwater from strongly reducing area of the Hetao Plain (Li et al., 2013, 2015; Wang et al., 2014a, 2015). Results showed that diverse microorganisms could be involved in As release and transformation in the Hetao Plain. The predominant populations in strong reducing conditions area of the Hetao Plain included *Acinetobacter, Pseudomonas, Psychrobacter*, and *Alishewanella* (Wang et al., 2014a; Li et al., 2015). These populations were reported to be related to As resistance, As reduction and oxidation, and iron reduction. As one of biggest agricultural areas in China, the subsurface hydrology in Hetao Plain has been largely affected by agricultural irrigation and drainage activities. Previous studies showed that drainage and irrigation would change natural biogeochemical processes and substantially affect As release in groundwater aquifers of the Hetao Plain (He, 2010; Guo et al., 2011). However, the effects of agricultural irrigation on *in situ* microbial diversity and community structure in high As groundwater have yet to be fully understood.

In the present study, we characterized and compared the microbial communities in groundwater along the agricultural drainage channels in Hetao Plain by Illumina sequencing. The objectives of this study were to (1) investigate the As geochemistry and the microbial community structure; (2) compare the microbial communities of high As groundwater in agricultural irrigation areas with those in strongly reducing high As groundwater of other areas at Hetao Plain; and (3) evaluate the key geochemical factors shaping the microbial community structures in high As groundwater aquifers in the agricultural irrigation area.

## Materials and Methods

### Site Description

**FIGURE 1:**
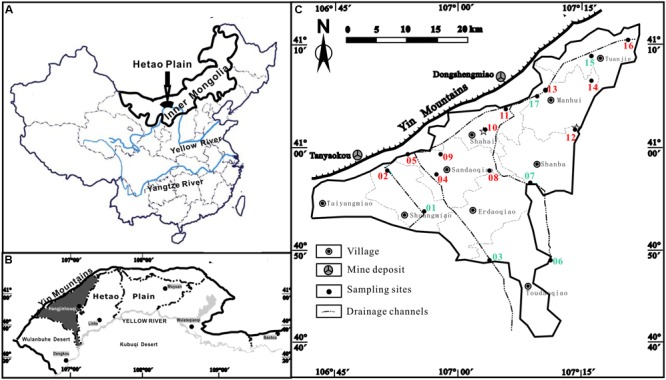
**Sampling sites of groundwater samples along the drainage channels in the present study. (A)** The Hetao Plain in China; **(B)** Hangjinhouqi County in the Hetao Plain; **(C)** groundwater sampling sites in Hangjinhouqi. 01–17 represented samples IC01–IC17. Green ones were low As samples with As concentrations lower than 10 μg/L. Red ones were high As samples with As concentrations higher than 10 μg/L. All high As samples except 05 and 13 presented ratios of As(III) and total As higher than 0.5.

The Hetao Plain (40°10′–41°20′N, 106°10′–109°30′E) is one of the Mesozoic-Cenozoic faulted basins located in the western part of Inner Mongolia. It is along the northern bank of the Yellow River, and is bounded to the north by the Yin Mountains and to the west by the Wulanbuhe Desert, and with lacustrine plain in the central part (**Figures 1A,B**). Groundwater in the Hetao Plain is discharged mainly by vertical evapotranspiration and pumping and recharged by irrigation. Our case study was carried out in Hangjinhouqi County (**Figures 1B,C**) where endemic arseniasis is most serious in the Hetao Plain, with about 76 thousands local residents exposed under As-rich groundwater (Deng, 2008). In Hangjinhouqi County, four main drainage channels (three SE–NW and one SW–NE orientated) have been used to drain redundant irrigational water and to lower the local groundwater table (**Figure 1C**).

### Groundwater Sampling and Geochemical Analysis

Groundwater samples along the agricultural drainage channels were collected from 17 domestic wells of seven arseniasis-affected villages in Hangjinhouqi County, of which sample IC12 and IC16 had been collected for the study of methanogens in high As groundwater in a previous study (Wang et al., 2015). Sampling locations were shown in **Figure 1**. Wells were pumped to get stabilized oxidation-reduction potential (ORP) values (commonly 10–15 min) prior to groundwater sampling. Microbial samples were collected by filtering of approximately 10 L fresh groundwater through 0.2-μm filters (Millipore), the filters were immediately packed into 50 mL sterile tubes, and then frozen in dry ice. All samples were transported to the laboratory on dry ice and then maintained at -80°C until further analysis. The geochemical parameters including temperature, pH, conductivity (COND), and ORP, were measured *in situ* using a multiple parameter water quality meter (Horiba, Japan). H_2_S, ${\mathrm{NH}}_{4}^{+}$, and Fe(II) were determined using a portable spectrophotometer (HACH, DR890) according to the manufacture’s protocols.

Sampling methods for ions and As speciation analyses were from Deng (2008). Briefly, groundwater samples were filtered through 0.45 μm mixed cellulose ester membranes and then the filtrates for cation analysis were acidified to pH < 2. Water samples were filtered through a disposable syringe joined with an anion exchange cartridge (Supelco, USA) to separate soluble arsenite [As(III)] and arsenate [As(V)] species, which were subsequently measured using liquid chromatography-atomic fluorescence spectrometry (LC-AFS-9700, Haiguang, China). Measurement of anions including ${\mathrm{NO}}_{3}^{–}$ and ${\mathrm{SO}}_{4}^{2–}$ was made with ion chromatography (DX-120, Dionex, USA). Major cations were determined with ICP-AES (IRIS Intrepid II XSP). Iron species were determined by the 1,10-phenanthroline-based assay which was described in our previous study (Li et al., 2013). Total organic carbon (TOC) was measured using a TOC analyzer (Vario MICRO cube, Elemental, Germany). All samples were run in triplicate and then averaged.

### DNA Extraction, PCR, and Illumina Sequencing

For the analysis of microbial community composition, the filtered membranes were cut into small pieces under aseptic conditions and used for DNA extraction by the FastDNA SPIN Kit for Soil (MP Bio, USA) with a final elution in 80 μL deionized water. The universal forward primer 515 (5′-GTG CCA GCM GCC GCG GTA A-3′) and reverse primer 806 (5′-GGA CTA CCA GGG TAT CTA AT-3′) barcoded with a 12-base Golay code, which could cover a wide diversity of both archaea and bacteria (Bates et al., 2011), were used to amplify the microbial 16S rRNA gene V4 region. The 25 μL PCR reaction mix consisted of 2.5 μL of Takara 10× Ex Taq Buffer, 2 μL of dNTP Mix (2.5 mM each), 0.7 μL of Roche bovine serum albumin (20 mg/mL), 0.125 μL of Ex Taq DNA polymerase (Takara, 5 U/μL), 0.5 μL of 10 μM primer 515F, 0.5 μL of 10 μM barcode primer 806R, 1 μL of template DNA, and 17.675 μL of PCR grade water. The PCR amplification condition was: initial denaturation at 95°C for 3 min, followed by 25 cycles of 95°C for 30 s, 50°C for 30 s, and 72°C for 45 s, and a final extension at 72°C for 10 min. All samples were run in triplicate and then pooled together. Successful amplifications were confirmed by E-Gel (Invitrogen, USA) electrophoresis. PCR products were purified using the Agencourt AMPure XP PCR purification system (Beckman Coulter, Brea, CA, USA). The purified amplicons were quantified using the Qubit dsDNA HS assay and the size of the amplicons was determined using a Bioanalyzer with Agilent DNA 1000 chips (Agilent Technologies, Santa Clara, CA, USA). Amplicons were pooled (25 ng per sample) and the mixed suspensions were then subjected to paired-end (PE) sequencing by an Illumina MiSeq 2000 instrument at the Yale Center for Genome Analysis.

### Data Analysis

Principal component analysis (PCA) between As concentrations and other geochemical parameters were conducted using CANOCO for windows version 4.5. Hierarchical cluster analysis was performed with the “vegan” package in RStudio^1^ using unweighted pair-group method with arithmetic means (UPGMA) and Euclidean distance measure. SPSS 20 software was used to analyze the significant differences (*p* ≤ 0.05) among different geochemical characteristics and microbial diversity.

The PE reads generated from Illumina MiSeq sequencing were assembled using FLASH (Fast Length Adjustment of Short reads) according to index sequence (Magoč and Salzberg, 2011). All sequences with ambiguous base calls were discarded. The PE sequences were also discarded if a mismatch was observed in the assembly. The output data was further analyzed using QIIME (quantitative insights into microbial ecology) v1.7.0. Operational taxonomic units (OTU) picking was performed using a “closed-reference” protocol. The most abundant sequence in each cluster was chosen to represent the cluster. All representative sequences were aligned with PyNAST. Chimeric sequences were identified with Chimera Slayer and excluded. Sequences were clustered against the Greengenes database^2^ at 97% identity using uclust. Following assignment, 12,000 successfully assigned sequences from each sample were chosen at random to allow for downstream analyzes and cross-sample comparison. Alpha diversity analysis involving rarefaction curves, Chao1 and Shannon diversity (representing estimated OTU numbers and microbial diversities in the community, respectively) indices and beta diversity analysis were generated using OTU picking result in QIIME software.

Multivariate community analysis including principal coordinate analysis (PCoA) and co-inertia analysis was performed with the ade4 package (Chessel et al., 2004; Dray and Dufour, 2007) within R programming environment^3^ using normalized OTU data. Correlated geochemical variables were removed from the analyzed table. Besides, some geochemical variables were made Log_10_ transformation to reach normal distribution before analysis. SIMPER (similarity percentage) analysis (Clarke and Warwick, 1994) was conducted to rank the top 10 OTUs that contributed to the dissimilarity index between high As groundwater samples and low As groundwater samples. The DNA sequences were deposited to the Short Read Archive database at NCBI (Accession number: SRR4996298).

## Results and Discussion

### Groundwater Geochemistry

**FIGURE 2:**
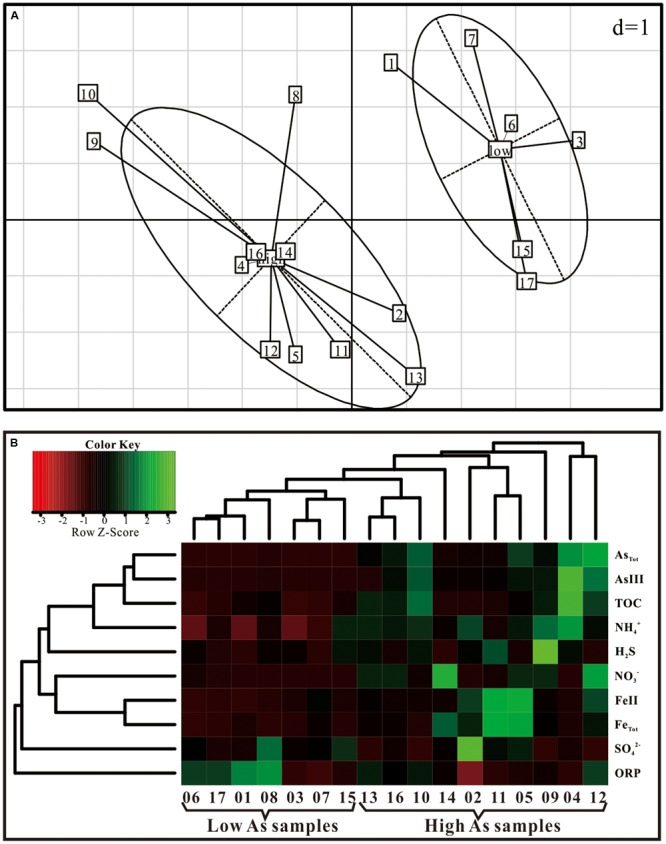
**Associations between geochemical parameters and samples of high (>50 μg/L) versus low (<10 μg/L) As concentrations by (A)** PCA plots based on As concentrations and **(B)** hierarchical cluster analysis.

Arsenic concentrations of groundwater in Hangjinhouqi County were generally elevated along the agricultural drainage channels in the front of Yin Mountains (**Figure 1**; **Table 1**). This was consistent with the results by He (2010) that As in soils or sediments could be leached into groundwater with the irrigation return flow. The 17 groundwater samples were divided into two groups with the distinctive geochemical characteristics according to the PCA (**Figure 2A**) and Hierarchical clustering results (**Figure 2B**). The first group (low As group) contained six groundwater samples which were characterized with low concentrations of As (As < 10 μg/L) and relatively low TOC and ${\mathrm{NH}}_{4}^{+}$. The other group (high As group) included 11 samples with high concentrations of As (As > 10 μg/L), TOC and ${\mathrm{NH}}_{4}^{+}$. Arsenic concentrations of those high As groundwater samples were from 50 to 1089 μg/L and As(III) was the dominant species in most of high As groundwaters, with ratios of As(III) to As_Tot_ ranging from 0.04 to 0.93 (**Table 1**). The concentrations of TOC lay in the range 1.9–18.4 mg/L in high As groundwater samples and 0–4.3 mg/L in low As samples. ${\mathrm{NH}}_{4}^{+}$ concentrations varied from 1.94 to 6.68 mg/L in high As groundwater samples and from 0 to 3.87 mg/L in low As groundwater samples.

**Table 1:** Geochemistry characteristics of groundwater samples along the drainage channels.

Sample no.	As_Tot_ (μg/L)	AsIII/As_Tot_	FeII (mg/L)	Fe_Tot_ (mg/L)	${\mathrm{NO}}_{3}^{–}$ (mg/L)	${\mathrm{SO}}_{4}^{2–}$ (mg/L)	${\mathrm{NH}}_{4}^{+}$ (mg/L)	H_2_S (μg/L)	TOC (mg/L)	pH	COND (s/m)	ORP (mv)
IC01	2	0.37	0.01	0.28	0.00	200.05	0.00	0	3.3	7.43	0.24	194
IC02	160	0.74	0.69	1.09	1.01	1458.10	4.75	7	1.9	8.05	2.45	-138
IC03	2	0.50	0.14	0.15	0.00	268.15	0.08	4	0	7.86	0.99	-60
IC04	991	0.93	0.14	0.33	0.00	222.60	6.68	8	18.4	7.59	0.35	-46
IC05	640	0.40	1.25	2.26	9.12	527.78	3.53	3	7.1	6.92	0.46	-22
IC06	2	0.50	0.01	0.03	1.87	357.79	0.08	4	0	7.95	1.14	114
IC07	2	0.50	0.02	0.11	0.00	83.73	0.05	3	4.3	8.05	0.46	-65
IC08	50	0.68	0.09	0.14	0.00	876.48	1.94	4	3.9	7.26	0.85	216
IC09	360	0.74	0.25	0.61	9.71	35.41	5.73	35	6.9	6.7	0.23	-187
IC10	745	0.64	0.22	0.22	2.41	33.51	4.28	9	11.7	7.02	0.17	-190
IC11	173	0.75	1.24	2.34	2.25	441.59	1.95	16	2.4	7.99	0.25	-50
IC12^∗^	1089	0.54	0.73	0.87	19.42	60.85	3.20	1	9.3	8.45	0.40	-278
IC13	335	0.04	0.27	0.50	8.53	84.84	3.88	8	7.3	8.31	0.30	76
IC14	152	0.75	0.20	1.60	22.17	296.33	2.34	0	1.9	8.03	0.49	5
IC15	10	0.81	0.16	0.18	0.00	615.12	3.87	9	2.9	7.73	1.80	-30
IC16^∗^	416	0.53	0.23	0.51	8.83	252.29	3.53	3	6.9	8.02	0.36	26
IC17	9	0.62	0.03	0.13	0.88	172.78	1.88	1	1.2	6.73	0.34	119

*Samples in the shaded part are low arsenic samples (As < 10 μg/L).*

*^∗^Samples were referred in our previous study (Wang et al., 2015).*

Arsenic concentrations of groundwater samples showed positive correlations with ${\mathrm{NH}}_{4}^{+}$ and TOC (**Table 2**; **Supplementary Figure S1**). ${\mathrm{SO}}_{4}^{2–}$ and Fe(II) concentrations in this study ranged from 35.41 to 1458.10 mg/L and 0.01 to 1.25 mg/L, respectively, showing no obvious correlations with As concentrations (**Table 2**). These results were different from those in our previous study of samples from strongly reducing areas in Hetao Plain that As concentrations showed negative correlations with the concentrations of ${\mathrm{SO}}_{4}^{2–}$ and positive correlations with the concentrations of Fe(II) (Li et al., 2015). Considering the positive correlation among As, ${\mathrm{NH}}_{4}^{+}$, and TOC, these results suggest that groundwater samples collected along the drainage channels might present distinct biogeochemical characteristics and redox properties. Agricultural activities such as N-fertilizer application and irrigation might also play important roles in As release and mobilization (Busbee et al., 2009; Mayorga et al., 2013). The reasonable explanation might be that the irrigation and drainage processes could leach As and dissolved organic matter from surficial sediments to the underlying aquifer, which caused the biogeochemical changes (He, 2010).

**Table 2:** Correlation between geochemical characteristics.

	AsIII	As_Tot_	AsIIIr	FeII	Fe_Tot_	FeIIr	${\mathrm{NO}}_{3}^{–}$	${\mathrm{SO}}_{4}^{2–}$	${\mathrm{NH}}_{4}^{+}$	H_2_S	TOC	COND
As_Tot_	0.926ˆ**											
AsIIIr	0.437ˆ*	0.286										
FeII	0.123	0.253	0.209									
Fe_Tot_	0.012	0.097	0.189	0.889ˆ**								
FeIIr	0.243	0.326	0.230	0.232	-0.106							
${\mathrm{NO}}_{3}^{–}$	0.013	0.100	-0.220	-0.008	0.264	-0.345						
${\mathrm{SO}}_{4}^{2–}$	-0.270	-0.309	-0.039	0.162	0.295	-0.150	0.520ˆ*					
${\mathrm{NH}}_{4}^{+}$	0.705ˆ**	0.688ˆ**	0.568ˆ**	0.106	0.001	0.295	-0.060	-0.130				
H_2_S	0.086	0.039	0.025	0.004	0.104	-0.127	0.487ˆ*	0.333	0.318			
TOC	0.892ˆ**	0.840ˆ**	0.284	-0.083	-0.173	0.142	0.001	-0.313	0.709ˆ**	0.161		
COND	-0.351	-0.413	0.065	0.141	0.081	0.243	-0.094	0.629ˆ**	-0.107	-0.080	-0.459ˆ*	
ORP	-0.092	-0.007	-0.291	-0.362	-0.248	-0.335	0.454ˆ*	0.171	-0.222	0.140	0.041	-0.335

*^∗^*p* < 0.05, ^∗∗^*p* < 0.01.*

### Microbial Richness and Diversity

The microbial community diversities and phylogenetic structure of the 17 groundwater samples were analyzed by Illumina sequencing. A total of 509,554 full length V4 tags were obtained after removal of low quality and chimeric sequences and 24,938 OTUs were obtained at a distance of 0.03. A variety of taxa were observed at the 97% OTU level, with 329–2823 observed OTUs and 0.64–0.84 coverage values (**Table 3**). The abundance of OTUs was observed to be higher in the low As groundwater samples than in high As samples (**Table 3**). The rarefaction curves generated by QIIME were normalized to the minimum number (17,000) of sequences. The Chao1 and Shannon diversity ranged from 465.26 to 3420.42 and 4.08 to 9.32, respectively (**Table 3**), showing higher values in low As groundwater samples (**Table 3**). These results implied that high As groundwater samples presented lower microbial diversities which was consistent with our result of the 16S rRNA gene clone library (Li et al., 2013). Compared with groundwater samples from strongly reducing areas of Hetao Plain (Li et al., 2015), samples collected along the agricultural drainage channels showed much higher microbial diversities, implying that irrigation and drainage activities might affect local geochemical environment and thus enhance the diversity of microorganisms.

**Table 3:** Alpha diversity indices at the 97% OTU level of groundwater samples.

Samples	Chao1	OTUs	Coverage of the observed OTUs (%)	PD_whole_tree	Shannon
IC01	1886.71	1355	0.72	97.42	6.44
IC02	2164.95	1567	0.72	120.65	7.12
IC03	2594.75	1849	0.71	142.27	8.22
IC04	1834.44	1234	0.67	93.12	6.18
IC05	2286.85	1524	0.67	121.47	7.82
IC06	2330.70	1657	0.71	126.84	6.38
IC07	2171.53	1426	0.66	110.88	5.99
IC08	996.21	705	0.71	43.24	6.20
IC09	465.26	391	0.84	15.44	4.16
IC10	497.84	329	0.66	16.77	4.08
IC11	2744.99	1767	0.64	129.56	6.83
IC12	1912.20	1484	0.78	110.13	7.85
IC13	3276.19	2394	0.73	171.60	8.78
IC14	1588.44	1148	0.72	86.22	6.30
IC15	2766.27	2121	0.77	162.58	9.19
IC16	1810.62	1164	0.64	85.24	6.07
IC17	3420.42	2823	0.83	196.72	9.32

*Samples in the shaded part are low arsenic samples (As < 10 μg/L).*

### Microbial Compositions in High As Groundwater

Microbial community compositions at phylum level showed no distinct difference between high and low As groundwater samples (**Supplementary Figure S2**). Illumina results of the 17 groundwater samples indicated that the bacterial community was composed of 62 phyla (percentages lower than 0.01% were not calculated). Of these, only nine phyla dominated each community (**Supplementary Figure S2**), among which Proteobacteria was the most dominant group (32.02–86.50%), followed by Firmicutes (0.16–18.48%), Actinobacteria (0.34–12.08%), and Nitrospirae (0–22.64%). Within phylum Proteobacteria, Gammaproteobacteria (6–83%), and Betaproteobacteria (5–39%) were the most abundant (**Supplementary Figure S2**). The Alphaproteobacteria and Deltaproteobacteria accounted for 0–17% and 0–23% of the total Proteobacteria, respectively. The class Epsilonproteobacteria displayed a low rate of occurrence and was only abundant in two high As groundwater samples IC02 and IC14 (**Supplementary Figure S2**). Archaeal populations accounted only for 0.01–8.69% of the microbial populations. All detected archaeal populations belonged to the phyla Euryarchaeota and Crenarchaeota which represented by the classes of Parvarchaea, Methanomicrobia, and Methanobacteria. Members of the Methanomicrobia and Methanobacteria retrieved at high As groundwater samples were mostly affiliated with methane-producing archaea *Methanocorpusculum, Methanospirillum* and SAGMEG-1 which have previously been detected in marine sediments and sludge (Iino et al., 2009; Barbier et al., 2012). These methanogenic populations might provide even stronger reducing conditions and thereby accelerate As release in groundwater aquifers in the Hetao Plain (Wang et al., 2015). However, irrigation and drainage activities in agricultural areas could bring more oxygen and the resulting oxidizing conditions might inhibit the activities of methanogenic populations.

**FIGURE 3:**
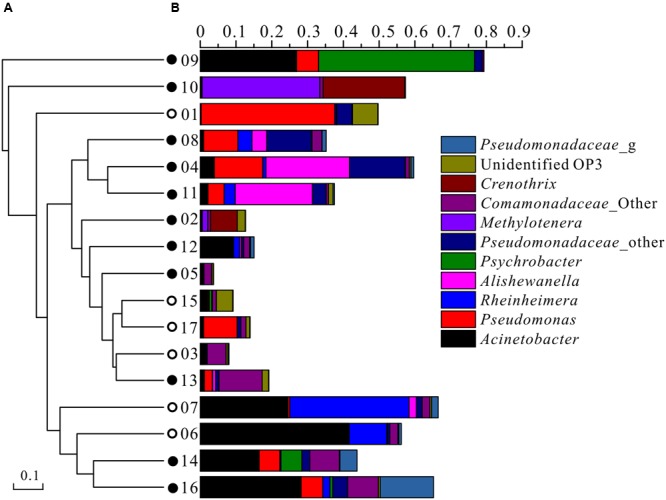
**Microbial community compositions of groundwater samples along the drainage channels.** Solid circles represented high As samples while open circles represented low As samples. **(A)** UPGMA cluster tree based on chord distance using genus data; **(B)** microbial compositions at the genus or family level. The legend showed the top 11 OTUs (at the 97% level).

At the genus level, the top 10 taxonomic OTUs that contributed to the dissimilarities between high and low As groundwater samples were identified by SIMPER analysis (**Table 4**). The average abundances of *Alishewanella, Psychrobacter*, Pseudomonadaceae, *Methylotenera*, Comamonadaceae, and *Crenothrix* were elevated in high As groundwater samples than in low As samples, while *Rheinheimera* and unidentified OP3 presented higher abundance in low As groundwater samples than high As samples (**Figure 3B**; **Table 4**). Although *Acinetobacter* and *Pseudomonas* had been reported to be correlated with As metabolism (Anderson and Cook, 2004; Chang et al., 2009; Freikowski et al., 2010), these two populations appeared with slightly higher percentages in low As groundwater samples but dominated in both high (0.2–28.2% and 0.1–13.6%, respectively) and low (0.2–41.4% and 0.2–37.3%, respectively) As samples in the present study. This might be due to the wide occurrence of these two kinds of bacteria in nature, and some of their isolates are capable of tolerating high concentrations of As. These results were mostly consistent with those of our previous studies in the strongly reducing area conducted with traditional sequencing and 454 pyrosequencing methods (Li et al., 2013, 2015). Predominant populations in high As groundwater samples such as *Alishewanella* and *Psychrobacter* were previously isolated from industrial eﬄuent or groundwater aquifers, showing the capability of hyper arsenite tolerance or arsenate reduction (Liao et al., 2011; Jain et al., 2014). In some recent studies, species of *Alishewanella* were also identified as denitrifiers (Kolekar et al., 2013; Liu et al., 2015). *Methylotenera* species were reported as facultative methylotrophs and could be involved in the carbon and nitrogen metabolic pathways (Mustakhimov et al., 2013; Chistoserdova, 2014). The sample with the highest concentration of TOC presented the highest abundance of *Methylotenera*. *Crenothrix* was documented as one kind of iron oxidizing bacteria in groundwater (Kang and Liu, 2013; Thapa Chhetri et al., 2014). Besides, *Crenothrix* was also reported as a methane oxidizer with a unique methane monooxygenase (Stoecker et al., 2006). These dominant populations in high As groundwater indicated that As release in groundwater along agricultural drainage channels was correlated with microbial carbon, nitrogen, and iron reactions. Different from our previous studies with samples from the natural reducing area (Li et al., 2013, 2015), many oxidizing microbial populations such as *Methylotenera* and *Crenothrix* were found in the present study, indicating the possible influence of geochemical difference on microbial composition. The dominant populations in this study were also different from those in high As groundwater aquifer in Bangladesh (such as *Hydrogenophaga* and *Acidovorax*; Sutton et al., 2009), West Bengal (such as *Agrobacterium–Rhizobium, Ochrobactrum, Anoxybacillus*, and *Paenibacillus*; Sarkar et al., 2015), and Vietnam (Lawati et al., 2012). These community differences might be influenced by different geochemical conditions such as ORP (Sutton et al., 2009) and dissolved organic carbon (Lawati et al., 2012).

**Table 4:** Top 10 OTUs (at the 97% level) for dissimilarity between high As groundwater samples and low As groundwater samples.

Taxon	Family/genus	Contribution 1 (%)	av.high^a^ (%)	av.low^b^ (%)
Gammaproteobacteria	*Acinetobacter*	7.14	8.18	11.71
Gammaproteobacteria	*Pseudomonas*	5.97	4.41	8.01
Gammaproteobacteria	*Rheinheimera*	6.01	1.10	7.41
Gammaproteobacteria	*Alishewanella*	4.36	4.59	0.44
Gammaproteobacteria	*Psychrobacter*	6.50	4.57	0.12
Gammaproteobacteria	Pseudomonadaceae	2.41	3.84	1.32
Betaproteobacteria	*Methylotenera*	4.96	3.16	0.03
Betaproteobacteria	Comamonadaceae	1.70	3.58	2.04
Gammaproteobacteria	*Crenothrix*	3.53	2.76	0.02
Gammaproteobacteria	Unidentified OP3	1.24	0.64	2.33

*^a^Average abundance of each OTU in high As samples. ^b^Average abundance of each OTU in low As samples.*

### Microbial Community Structure in Relation to Geochemistry

**FIGURE 4:**
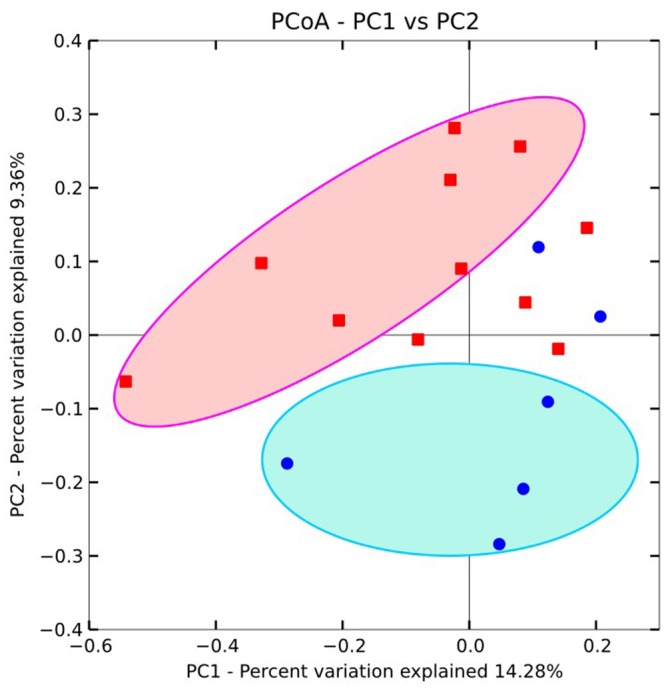
**Principal component analysis for the total microbial communities in groundwaters from the Hetao Plain.** Red squares: As concentrations > 10 μg/L; while blue circles: As concentrations <10 μg/L.

PCoA plots were used to visualize the relationships among microbial communities using default beta diversity metrics of unweighted Unifrac. Geochemical parameters including pH, ORP, As, Fe, ${\mathrm{SO}}_{4}^{2–}$, ${\mathrm{NH}}_{4}^{+}$, and TOC were used to calculate the possible influence on microbial differences. Results showed that microbial communities could be generally divided into two groups according to different As concentrations (**Figure 4**). A similar result was also obtained with UPGMA cluster tree analysis which was based on Bray-Curtis dissimilarity at the 97% similarity OTU level (**Figure 3A**). These results implied that As was an important factor shaping the microbial community structure in groundwater of agricultural irrigation area.

**FIGURE 5:**
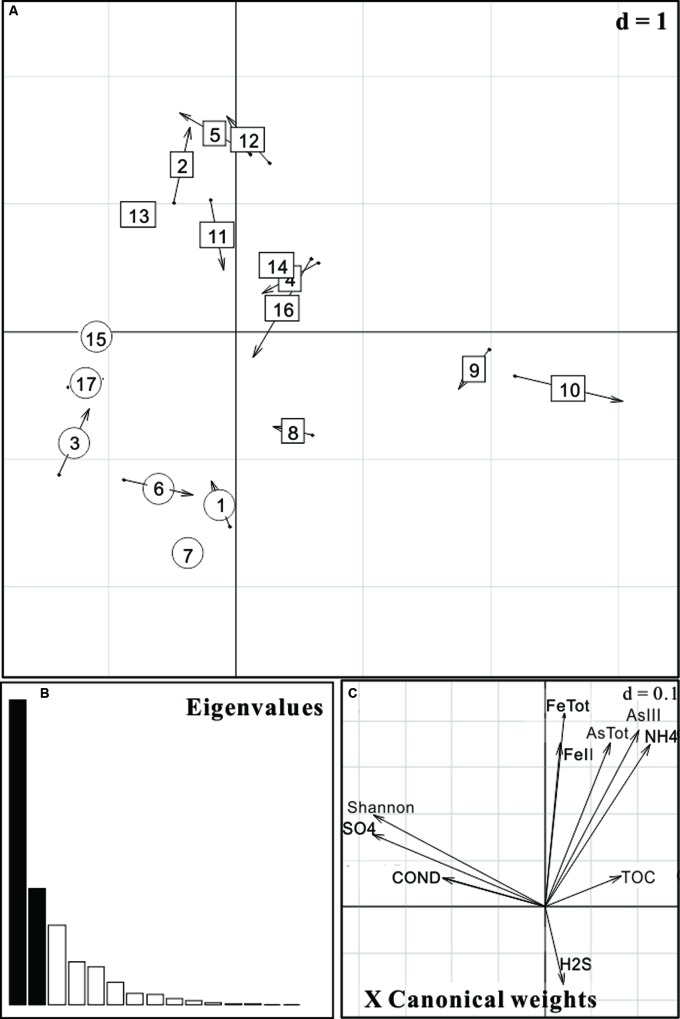
**Co-inertia analysis (CIA) of microbial community and geochemical parameters of 17 groundwater samples from the Hetao Plain. (A)** Samples projected on the first two axes; **(B)** Eigenvalues; **(C)** Main geochemical vectors that affect sample ordination in the CIA. As concentration was used as a factor. Circular ones represent low As samples (As < 10 μg/L) while rectangular ones represent high As samples (As > 10 μg/L).

Co-inertia analysis was carried out in order to reveal the relative importance of geochemical vectors that affect microbial community structures. The arrow head and end represented the position of sample according to the geochemical ordination and microbial ordination, respectively (**Figure 5**). As shown in **Figure 5B**, the first two axes explained 81% of the total environmental variability. Results revealed that high As groundwater and low As groundwater samples were significantly different (*p* value < 0.001) in community structures (**Figure 5A**) and geochemical variables had a significant influence on their microbial community compositions. In addition to As concentration, geochemical variables such as TOC, ${\mathrm{NH}}_{4}^{+}$, Fe, and ORP might also affect the composition (**Figure 5C**). These important geochemical factors shaping the microbial community structure were different from those of strong reducing area in the Hetao Plain which were characterized with high concentrations of Fe(II), H_2_S, and ${\mathrm{SO}}_{4}^{2–}$.

## Conclusion

Arsenic concentrations in groundwater samples along agricultural drainage channels of the Hetao Plain were positively correlated with concentrations of TOC and ${\mathrm{NH}}_{4}^{+}$. Illumina sequencing of the 16S rRNA genes demonstrated that a diverse array of microorganisms existed in the high As groundwater aquifers which were mainly composed of Proteobacteria, Firmicutes, Actinobacteria, and Nitrospirae. The dominant microbial populations were distinctly different between the high and low As groundwater samples. The predominant groups in high As samples were *Alishewanella, Psychrobacter, Methylotenera*, and *Crenothrix*. Microbial groups such as *Rheinheimera* and unidentified OP3 were much more predominant in low As samples. These dominant populations in high As groundwater were previously reported to be correlated with microbial carbon, nitrogen, and iron reactions. Oxidizing populations such as *Methylotenera* and *Crenothrix* were found in samples along agricultural drainage channels, which was different from our previous studies in samples from other areas under strongly reducing conditions. Statistic results indicated that microbial community structures were different between high and low As groundwater samples, under the impact of As concentrations as well as other geochemical variables including TOC, ${\mathrm{NH}}_{4}^{+}$, ORP, and Fe. Overall, the results of this study indicate that both the geochemistry and microbial community structures in groundwater agricultural areas are different from those in other As-rich aquifers of Hetao Plain, Inner Mongolia and other high As groundwater aquifers such as Bangladesh, West Bengal, and Vietnam.

## Author Contributions

YXW and PL designed experiments; YHW carried out experiments and wrote the manuscript; YHW and ZJ analyzed experimental results; AS assisted with sequencing data analysis; SW assisted with Illumina sequencing experiment; HLD, YXW, and PL revised the manuscript; JT and DW collected all samples needed in the study.

## Conflict of Interest Statement

The authors declare that the research was conducted in the absence of any commercial or financial relationships that could be construed as a potential conflict of interest.

## Footnotes

1 http://www.rstudio.com/

2 http://greengenes.lbl.gov

3 http://www.rstudio.com/

## Supplementary Material

FIGURE S1 **PCA ordination plots between environment variables and groundwater samples using CANOCO software.** Axis 1 and axis 2 account for 68% and 10.8% of the variance, respectively. Solid triangle ones represent high As samples, while hollow triangle ones represent low As samples.

FIGURE S2 **Relative abundance of OTUs showing the microbial community distribution at taxonomic level of (A)** bacterial phyla and **(B)** bacterial proteobacterial classes.
