# Supplementary figures and images for: Microbial Community of High Arsenic Groundwater in Agricultural Irrigation Area of Hetao Plain, Inner Mongolia
Source: Front Microbiol. 2016 Dec 6;7:1917. doi: 10.3389/fmicb.2016.01917 (PMC5138239; doi:10.3389/fmicb.2016.01917)

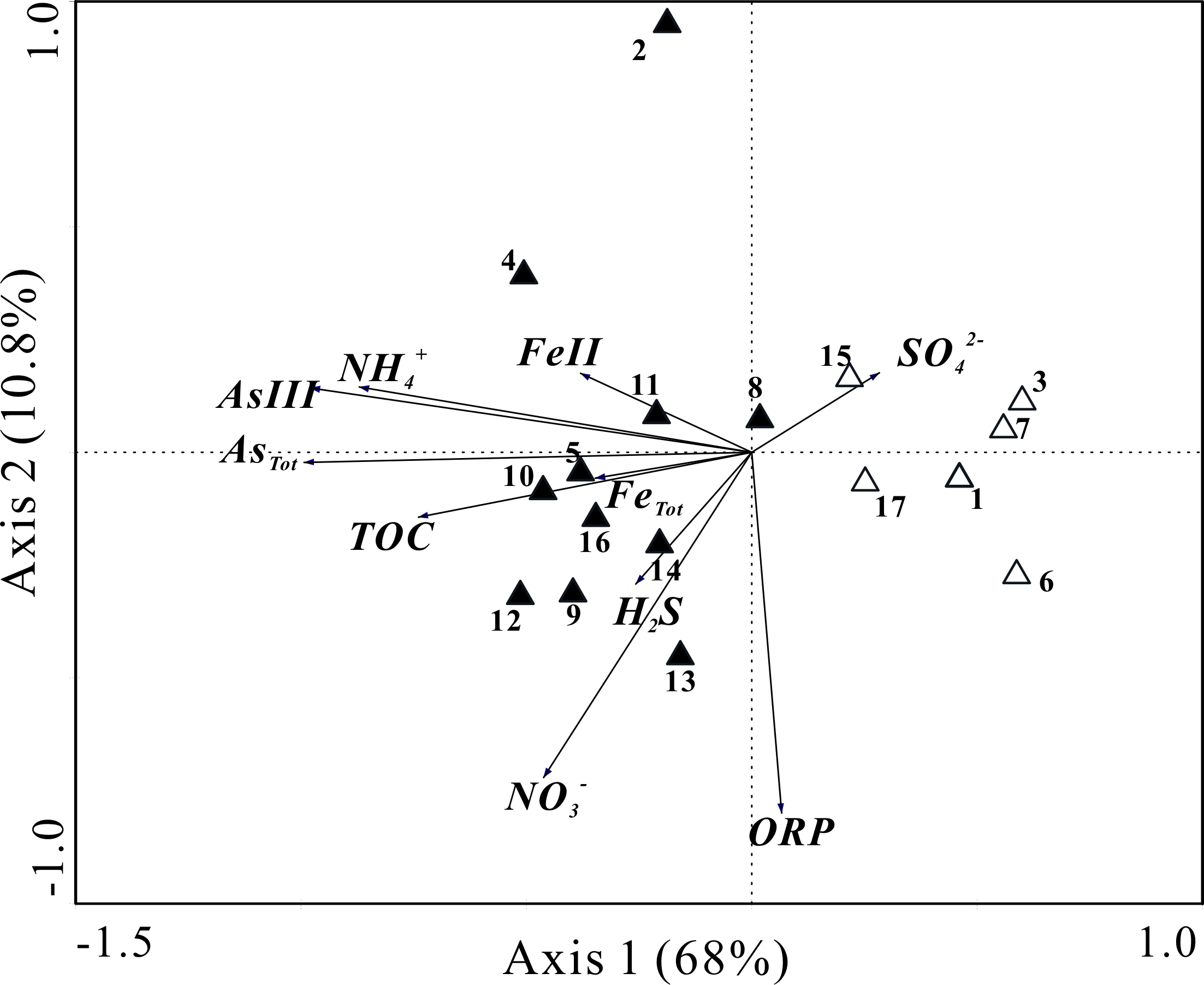

Supplement: FIGURE S1 — PCA ordination plots between environment variables and groundwater samples using CANOCO software. Axis 1 and axis 2 account for 68% and 10.8% of the variance, respectively. Solid triangle ones represent high As samples, while hollow triangle ones represent low As samples. [file Image_1.TIF]

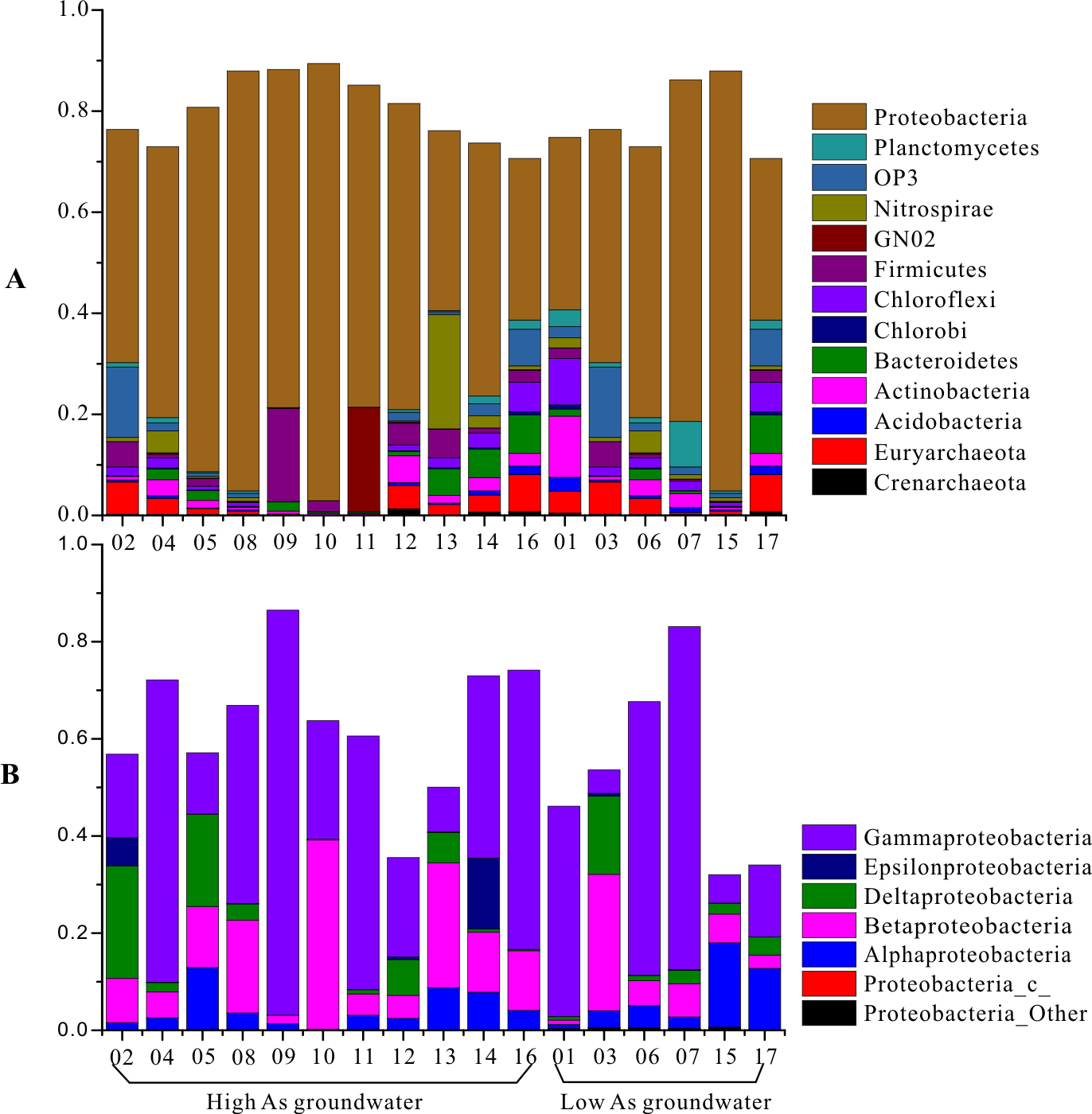

Supplement: FIGURE S2 — Relative abundance of OTUs showing the microbial community distribution at taxonomic level of (A) bacterial phyla and (B) bacterial proteobacterial classes. [file Image_2.TIF]
